# ^18^F-Sodium fluoride PET-CT visualizes disease activity in chronic nonbacterial osteitis in adults

**DOI:** 10.1093/jbmrpl/ziad007

**Published:** 2024-01-04

**Authors:** Anne T Leerling, Frits Smit, Zita Spӓth, Ana Navas Cañete, Lioe-Fee de Geus-Oei, Alina van de Burgt, Olaf M Dekkers, Wouter van der Bruggen, Natasha M Appelman-Dijkstra, Dennis Vriens, Elizabeth M Winter

**Affiliations:** Department of Internal Medicine, Division of Endocrinology, Leiden University Medical Center, Leiden, 2333 ZA, The Netherlands; Center for Bone Quality, Leiden University Medical Center, Leiden, 2333 ZA, The Netherlands; Department of Clinical Epidemiology, Leiden University Medical Center, Leiden, 2333 ZA, The Netherlands; Center for Bone Quality, Leiden University Medical Center, Leiden, 2333 ZA, The Netherlands; Department of Radiology, Section of Nuclear Medicine, Leiden University Medical Center, Leiden, 2333 ZA, The Netherlands; Department of Nuclear Medicine, Alrijne Hospital, Leiderdorp, 2353 GA, The Netherlands; Department of Internal Medicine, Division of Endocrinology, Leiden University Medical Center, Leiden, 2333 ZA, The Netherlands; Center for Bone Quality, Leiden University Medical Center, Leiden, 2333 ZA, The Netherlands; Center for Bone Quality, Leiden University Medical Center, Leiden, 2333 ZA, The Netherlands; Department of Radiology, Section of Nuclear Medicine, Leiden University Medical Center, Leiden, 2333 ZA, The Netherlands; Center for Bone Quality, Leiden University Medical Center, Leiden, 2333 ZA, The Netherlands; Department of Radiology, Section of Nuclear Medicine, Leiden University Medical Center, Leiden, 2333 ZA, The Netherlands; Department of Radiation Science and Technology, Delft University of Technology, Delft, 2628 CD, The Netherlands; Department of Nuclear Medicine, Alrijne Hospital, Leiderdorp, 2353 GA, The Netherlands; Department of Internal Medicine, Division of Endocrinology, Leiden University Medical Center, Leiden, 2333 ZA, The Netherlands; Department of Clinical Epidemiology, Leiden University Medical Center, Leiden, 2333 ZA, The Netherlands; Department of Nuclear Medicine, Slingeland Hospital, Doetinchem, 7009 BL, The Netherlands; Department of Internal Medicine, Division of Endocrinology, Leiden University Medical Center, Leiden, 2333 ZA, The Netherlands; Center for Bone Quality, Leiden University Medical Center, Leiden, 2333 ZA, The Netherlands; Center for Bone Quality, Leiden University Medical Center, Leiden, 2333 ZA, The Netherlands; Department of Radiology, Section of Nuclear Medicine, Leiden University Medical Center, Leiden, 2333 ZA, The Netherlands; Department of Internal Medicine, Division of Endocrinology, Leiden University Medical Center, Leiden, 2333 ZA, The Netherlands; Center for Bone Quality, Leiden University Medical Center, Leiden, 2333 ZA, The Netherlands

**Keywords:** osteitis, osteomyelitis, SAPHO, CNO, PET/CT, biomarker, bone turnover, bone inflammation

## Abstract

Chronic nonbacterial osteitis (CNO) is a rare disease spectrum, which lacks biomarkers for disease activity. Sodium fluoride-18 positron emission tomography/computed tomography ([^18^F]NaF-PET/CT) is a sensitive imaging tool for bone diseases and yields quantitative data on bone turnover. We evaluated the capacities of [^18^F]NaF-PET/CT to provide structural and functional assessment in adult CNO. A coss-sectional study was performed including 43 adult patients with CNO and 16 controls (patients referred for suspected, but not diagnosed with CNO) who underwent [^18^F]NaF-PET/CT at our expert clinic. Structural features were compared between patients and controls, and maximal standardized uptake values (SUV_max_ [g/mL]) were calculated for bone lesions, soft tissue/joint lesions, and reference bone. SUV_max_ was correlated with clinical disease activity in patients. Structural assessment revealed manubrial and costal sclerosis/hyperostosis and calcification of the costoclavicular ligament as typical features associated with CNO. SUV_max_ of CNO lesions was higher compared with in-patient reference bone (mean paired difference: 11.4; 95% CI: 9.4–13.5; p < .001) and controls (mean difference: 12.4; 95%CI: 9.1–15.8; p < .001). The highest SUV_max_ values were found in soft tissue and joint areas such as the costoclavicular ligament and manubriosternal joint, and these correlated with erythrocyte sedimentation rate in patients (correlation coefficient: 0.546; p < .002). Our data suggest that [^18^F]NaF-PET/CT is a promising imaging tool for adult CNO, allowing for detailed structural evaluation of its typical bone, soft-tissue, and joint features. At the same time, [^18^F]NaF-PET/CT yields quantitative bone remodeling data that represent the pathologically increased bone turnover and the process of new bone formation. Further studies should investigate the application of quantified [18F]NaF uptake as a novel biomarker for disease activity in CNO, and its utility to steer clinical decision making.

## Graphical Abstract

**Graphical Abstract:**
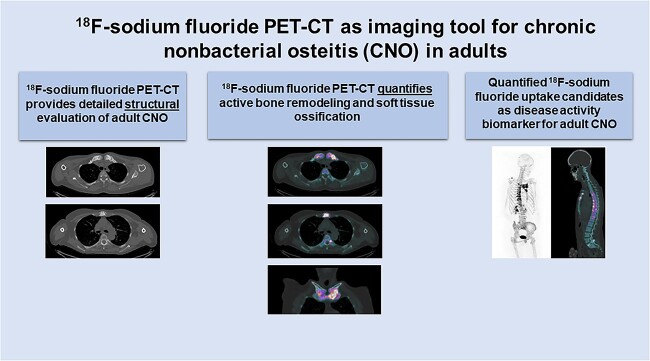

## Introduction

Chronic nonbacterial osteitis (CNO) is an inflammatory bone disease affecting children and adults. The CNO spectrum is heterogeneous, ranging from cases of isolated sterile bone inflammation to those with varying additional (extraskeletal) features such as arthritis, psoriasis, acne, and pustulosis palmoplantaris. In absence of aligned terminology, adult patients with CNO may currently be referred to as synovitis, acne, pustulosis, hyperostosis, osteitis (SAPHO) syndrome as well.^1^^,^^2^ In adult CNO, bone inflammation mainly occurs in the anterior chest wall (involved in 89%), followed by the spine, mandible, and incidentally, the peripheral skeleton.^3^ Typical structural imaging features include sclerosis, hyperostosis, ossification of soft tissue, ankylosis, and degeneration of the adjacent joints, which all gradually accumulate over time.^4^

Although the CNO disease course is generally chronic, inflammatory disease activity can vary over time and may be relapsing and remitting as well.^1^ The evaluation of disease activity, however, is highly challenging currently, as biochemical markers of inflammation are only increased in a minority of patients. With regard to imaging biomarkers, bone scintigraphy using technetium-99m radio-labeled hydroxymethylene diphosphonate ([^99m^Tc]Tc-HDP) visualizes the increased bone turnover resulting from active inflammation, but continues to demonstrate increased uptake despite clinical remission, suggesting an imprinting pattern.^5^ With regard to magnetic resonance imaging (MRI), the key CNO features of sclerosis and hyperostosis hamper the proper detection of bone marrow edema on T2-weighted images with fat suppression and short tau inversion recovery (STIR)-weighted images, and similarly prevents the assessment of areas of restricted diffusion on diffusion-weighted imaging.^6^^,^^7^

Sodium fluoride-18 positron emission tomography/computed tomography ([^18^F]NaF-PET/CT) is a widely used tool in metabolic bone disease,^8–10^ and poses distinct advantages compared with other nuclear imaging techniques. Compared with [^99m^Tc]Tc-HDP, it requires a shorter incubation time, shorter scanning time, and gives lower radiation exposure.^9–11^ Compared with a combination of [^99m^Tc]Tc-HDP bone scintigraphy with single positron emission computed tomography (SPECT/CT), it offers superior spatial resolution for anatomical orientation. In addition, [^18^F]NaF-PET/CT readily yields quantitative bone turnover signals that have shown to strongly correlate with clinical disease activity in other metabolic bone diseases, qualifying the imaging tool as a disease-monitoring instrument.^9^

This study therefore aimed to evaluate the application of [^18^F]NaF-PET/CT in adult CNO, both in providing structural assessments of CNO lesions, as well as functional evaluation via quantitative bone turnover parameters.

## Materials and methods

This study was approved by the medical-ethical review board associated with the Leiden University Medical Center. Written consent was obtained from all individuals for the pseudo-anonymized use of their data. The study conduct adhered to the Strengthening the Reporting of Observational Studies in Epidemiology (STROBE) guidelines.^12^

### Study population and design

A cross-sectional study was conducted at the Center for Bone Quality of the Leiden University Medical Center, a tertiary referral and expert center for CNO. Adult patients with suspected CNO referred between 2019 and 2022 were included in the study as, from this time, [^18^F]NaF-PET/CT was performed as part of standard care at presentation or during flare-ups. In absence of validated classification criteria for adult CNO, eventual diagnosis was expert-based on a combined clinical and imaging assessment of an experienced endocrinologist, radiologist, and nuclear imaging physician. Features regarded as indicative of CNO were chronic or relapsing–remitting inflammatory bone pain and sclerosis and hyperostosis on imaging, isolated or accompanied by extraosseous features, such as synovitis, skin manifestations, or other auto-inflammatory comorbidities. Importantly, quantified uptake on [^18^F]NaF-PET/CT did not affect diagnosis as these data were retrospectively obtained for the present study. Controls were individuals presenting with CNO-like complaints (mostly pain in the anterior chest wall) but without sufficient features for diagnosis or in whom another diagnosis was made. Individuals were excluded in case of an indefinite diagnosis, if [^18^F]NaF-PET/CT data were incomplete, or whenever [^18^F]NaF-PET/CT was performed after the initiation of therapy.

### [^18^F]NaF-PET/CT acquisition

Whole-body [^18^F]NaF-PET/CT was performed on a 5-ring Discovery MI PET/CT (GE Healthcare, Chicago, IL, USA). Data acquisition was performed approximately 30 minutes after intravenous Na^18^F administration of approximately 1 MBq/kg body weight. An emission scan was obtained using multiple bed positions with 50% overlap between bed positions and 40 seconds per bed position. Time-of-flight PET data were reconstructed using a 256 × 256 matrix, the point spread function and CT-based attenuation correction (120 kV, smart mA modulations with a noise index of 49.5 and a tube current ranging from 15 to 550 mA, 0.5-s rotation time).

### Analysis of structural imaging features on [^18^F]NaF-PET/CT

All [^18^F]NaF-PET/CT scans were assessed by 1 experienced nuclear physician (F.S.) and radiologist (A.N.C.) in a Picture Archiving and Communication System (PACS) following a systematic reporting format. Upon disagreement, eventual assessment was consensus-based. For each possible lesion site (clavicles, costae, manubrium, sternoclavicular joints, costoclavicular ligaments, costosternal joints, costochondral transitions, mandible, and spine), the degrees of sclerosis, hyperostosis, erosions, ankylosis, and [^18^F]NaF uptake were scored as appropriate (see Supplementary data (S1) for full systematic reporting format, including detailed definitions of abnormalities).

### Quantitative analysis of [^18^F]NaF-PET/CT

Volumes of interest (VOIs) to generate quantitative parameters of isotope uptake were determined on European Association of Nuclear Medicine Research GmbH (EARL)-compliant reconstructed PET images fused with low-dose CT. The VOIs were drawn using CT images, without blinding for patient or control status but with blinding for degree of uptake (fused PET images were not enabled during the drawing of VOIs). In patients, up to 2 areas of abnormal sclerosis in bone were selected to represent a “bone lesion.” Also, up to 2 areas with soft tissue abnormalities in the anterior chest wall (ossification, ankylosis, degeneration; possibly in the sternoclavicular joints, costosternal joints, costochondral transitions, costoclavicular ligaments, or manubriosternal joint) were selected to represent “soft tissue/joint lesions.” If more than 2 diseased bone/soft tissue/joint areas were available, the 2 most pronounced areas were selected. In controls, 3 VOIs were drawn in the proximal clavicles and the manubrium sterni to represent control “bone lesions,” and 5 in the ipsilateral sternoclavicular joint, first costosternal joint, first costochondral transition, costoclavicular ligament, and the manubriosternal joint to represent control “soft tissue/joint lesions.” For both patients and controls, 2 VOIs were drawn in reference bones (ref-bone): the fifth vertebral body (Th5) and the proximal humerus, except in the case of confounding pathology in 1 of these locations (*n* = 3 and *n* = 1, respectively). From all VOIs, the maximum standardized uptake value (SUV_max_) in g/mL was calculated, which is the decay-corrected activity concentration of the radiopharmaceutical (in Bq/mL) standardized for administered activity (in Bq) and subject body weight (in g). This parameter was selected for its reproducibility and widespread use in clinical practice. Absolute mean SUV_max_ of bone and soft tissue/joint lesions plus the ratio between bone lesions/ref-bone were compared between patients and controls (overall and stratified per anatomical location).

### Clinical and biochemical data

The following ancillary data were obtained from the electronic health records for patients and controls: age, gender, height and weight, auto-inflammatory comorbidity, and current medication use. Clinical disease status was evaluated by a visual analog scale (VAS) for pain (VAS 0–10, with 0 representing no pain and 10 representing the worst imaginable pain) on the Brief Pain Inventory^13^ and biochemical values for inflammation and bone turnover (erythrocyte sedimentation rate [ESR], C-reactive protein [CRP], procollagen type 1N propeptide [P1NP], alkaline phosphatase [ALP], and C-terminal telopeptide of type 1 collagen [CTx]). The VAS scores and biochemical parameters were only included if collected within 8 weeks prior or after [^18^F]NaF-PET/CT.

### Statistical analysis

SPSS Statistics version 25 (IBM Corporation, Armonk, NY, USA) was used for statistical analyses. Data are presented as frequency of counts and percentages for categorical variables and mean ± SD or median (IQR) for continuous variables. Clinical and radiologic characteristics and SUV_max_ of bone and soft tissue/joint lesions were compared between patients and controls using chi-square tests (or Fisher’s exact test in case of ≥1 cells with expected frequencies <5) for proportions and categorical data, *t* test or 1-way ANOVA for parametric numerical data, and Mann-Whitney tests for nonparametric numerical data. The discriminatory capacity of individual radiologic features was evaluated with binary logistic regression; Haldane-Anscombe correction was applied in zero-cell cases.^14^ The number of bone and soft tissue/joint lesions and their SUV_max_ was correlated with clinical disease activity (biochemical parameters and VAS score for pain), age, and disease duration with Pearson’s correlation coefficient or Spearman’s rank test, as appropriate, in patients. Diagnostic capacities of mean SUV_max_ of bone and soft tissue/joint lesions were evaluated by multivariate logistic regression, adjusted for symptom duration. Cutoffs were optimized for specificity so as to minimize false-positives. The level of significance was set at p < .05.

## Results

**Figure 1:**
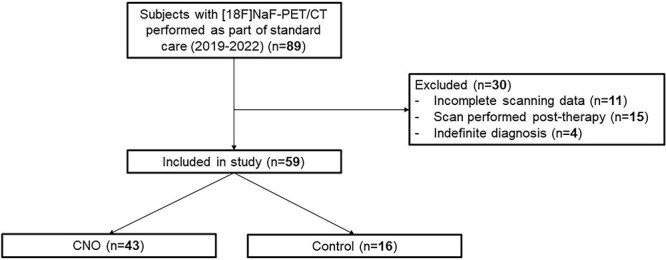
Overview of study inclusion. Abbreviations: CNO, chronic nonbacterial osteitis; [18F]NaF-PET/CT, sodium fluoride-18 positron emission tomography/computed tomography.

Eighty-nine individuals underwent [^18^F]NaF-PET/CT scanning between 2019 and 2022, of whom 43 patients with CNO and 16 controls could be included in the present study (Figure 1).

### Clinical characteristics of patients with CNO and controls

The clinical characteristics of patients and controls included for qualitative and quantitative analysis of [^18^F]NaF-PET/CT images are displayed in Table 1. Gender, body mass index, comorbidity, [^18^F]NaF incubation time, clinical symptoms, and medication use at the moment of scanning were all similar between patients and controls. Patients were slightly younger than controls (age: 45 ± 10 vs 49 ± 18 years). Controls were diagnosed with osteoarthritis (*n* = 7, 44%), functional complaints (*n* = 7, 44%), heterotrophic calcifications of the costal cartilage (*n* = 1, 6%), and a subchondral cyst (*n* = 1, 6%).

**Table 1:** Clinical characteristics of patients with CNO and controls.

	**CNO (*n* = 43)**	**Controls (*n* = 16)**	**p**
Gender, female, *n* (%)	39 (91)	14 (88)	.718
Age, y	45 ± 10	49 ± 18	.247
Body mass index, kg/m^2^	27 ± 6	26 ± 6	.874
Symptom duration, y	11 (7–19)	3 (2–13)	.006
[^18^F]NaF incubation time, min	41 ± 8	43 ± 12	.391
Skeletal CNO involvement pattern, *n* (%)			
Anterior chest wall	41 (95)	n.a.	
Spine	8 (19)	n.a.	
Additional (extraskeletal) features, *n* (%)			.976
Peripheral inflammatory arthritis	3 (7)	0 (0)	
Axial inflammatory arthritis / sacroiliitis	4 (9)	0 (0)	
PPP	14 (33)	3 (19)	
Psoriasis	6 (14)	1 (6)	
Other auto-inflammatory feature	2 (5)	3 (19)	
Medication use, *n* (%)			
None	16 (37)	6 (38)	.984
NSAIDs	24 (56)	7 (44)	
DMARDs	2 (5)	0 (0)	
TNF-α inhibitors	2 (5)	0 (0)	
Bisphosphonates	0 (0)	0 (0)	
Biochemical evaluation			
ESR, mm/h	11 (6–19)	12 (6–27)	.844
CRP, mg/L	3 (1–9)	1.7 (1–3)	.115
P1NP, μg/L	49 (26–62)	58 (41–68)	.357
CTx, μg/L	0.24 (0.17–0.36)	0.29 (0.17–0.35)	.781
ALP, U/L	80 (59–98)	84 (61–112)	.362
Clinical symptoms			
VAS average pain (0–10)	5 ± 2	5 ± 2	.682

Abbreviations ALP, alkaline phosphatase; CNO, chronic nonbacterial osteitis; CRP, C-reactive protein; CTx, C-terminal telopeptide of type 1 collagen; DMARD, disease-modifying anti-rheumatic drug; ESR, erythrocyte sedimentation rate; n.a., not applicable; NSAID, nonsteroidal anti-inflammatory drug; P1NP, procollagen 1 N-terminal propeptide; PPP, palmoplantar pustulosis; TNF-α, tumor necrosis factor alpha; VAS, visual analog scale (10 representing worst pain); [^18^F]NaF, sodium fluoride-18.

Data are shown as means ± SD, median (IQR), or *n* (%).

### Structural evaluation of CNO on [^18^F]NaF-PET/CT

Structural imaging characteristics as scored via standardized reporting formats for bone are depicted in Table 2. In patients, sclerosis and hyperostosis were frequently found in costae 1 and 2 (79% for both), manubrium (77% and 70%), and to a slightly lesser extent in the clavicle (70% and 33%). Bilateral involvement was most common for the costae (67% displaying sclerosis/hyperostosis on both sides). In controls, radiologic abnormalities were also detected, but bilateral sclerosis of the clavicle and hyperostosis of the clavicle or manubrium were exclusively seen in patients with CNO. For both patients and controls, increased [^18^F]NaF uptake was generally found in the presence of radiologic abnormalities, with the exception of costae 1 and 2 for controls; here, increased uptake was also seen without signs of osteitis (56% increased uptake, only 6% radiologic abnormalities). Bone features that best differentiated patients and controls were sclerosis, hyperostosis, and increased uptake of the manubrium, and also sclerosis and hyperostosis of costae 1 and 2 (but not increased uptake) (p value for odds ratio [OR] = .004 for manubrial hyperostosis, p value for OR < .001 for others). Also (tending towards) differential were bilateral clavicular sclerosis (OR, 20; 95% CI, 0.9–424; p = .058) and bilateral increased uptake (OR, 10; 95% CI, 1–81; p = .034). In general, spinal features were only seen in few patients (19% with sclerosis/hyperostosis compared to 6% in controls).

**Table 2:** Imaging characteristics of bone in patients with CNO and controls on [^18^F]NaF-PET/CT.

	**CNO (*n* = 43)**	**Controls (*n* = 16)**	**OR (95% CI)**	**p**
Clavicle Sclerosis Bilateral Hyperostosis Bilateral Increased [^18^F]NaF uptake Bilateral	30 (70) 14 (33) 20 (47) 8 (19) 31 (72) 17 (40)	7 (44) 0 (0) 0 (0) 0 (0) 8 (50) 1 (6)	3 (0.9–10) 20 (0.9–424) 35 (2–761) 9 (0.4–217) 3 (0.8–8) 10 (1–81)	.072 .058 .022 .161 .116 .034
Manubrium Sclerosis Hyperostosis Increased [^18^F]NaF uptake	33 (77) 32 (74) 30 (70)	1 (6) 0 2 (13)	50 (6–422) 102 (5–2280) 20 (4–104)	< .001 <.001 .004
Costae 1–2 Sclerosis/hyperostosis Bilateral Increased [^18^F]NaF uptake Bilateral	34 (79) 29 (67) 35 (81) 25 (58)	1 (6) 1 (6) 9 (56) 6 (38)	57 (7–488) 31 (4–259) 3 (0.9–12) 2 (0.7–8)	<.001 .002 .055 .163
Spine (involved in *n* = 8 patients) Sclerosis/hyperostosis Increased [^18^F]NaF uptake	8 (100) 7 (88)	1 (6) 2 (13)	3 (0.4–30) 2 (0.4–6)	.256 .545

Abbreviations: CNO, chronic nonbacterial osteitis; OR, odds ratio; [^18^F]NaF, sodium fluoride-18; [18F]NaF-PET/CT, sodium fluoride-18 positron emission tomography/computed tomography.

Data are presented as *n* (%).

Soft tissue/joint tissues (eg, joints, ligaments) were also evaluated for radiologic features (see Table 3). Abnormalities found in the sternoclavicular joints and manubriosternal joint did not discriminate between patients and controls. In contrast, calcification/beginning ankylosis with increased uptake of the costoclavicular ligament was almost exclusively seen in patients (58% vs 6% and 54% vs 0%; OR [95% CI], 21 [3–172] and 48 [2–1022], respectively). This was similar for radiologic involvement of the costosternal joints (77% in patients vs 6% in controls; OR, 57; 95% CI, 7–488; p < .001), but not for uptake at this location, which only tended to be higher in patients than in controls.

**Table 3:** Imaging characteristics of soft tissue and joints in patients with CNO and controls on [^18^F]NaF-PET/CT.

	**CNO (*n* = 43)**	**Controls (*n* = 16)**	**OR (95% CI)**	**p**
Sternoclavicular joint(s) Erosion Calcification Increased [^18^F]NaF uptake	14 (32) 0 (0) 12 (28)	3 (19) 1 (6) 2 (13)	2 (0.5–9) 0.8 (0.4–1) 3 (0.5–14)	.304 .362 .229
Manubriosternal joint Erosion Ankylosis (noncongenital) Increased [^18^F]NaF uptake	14 (33) 4 (9) 15 (35)	1 (6) 0 (0) 2 (13)	7 (0.9–60) 4 (0.2–115) 4 (0.8–19)	.068 .373 .107
Costoclavicular ligament(s) Calcification/ankylosis Increased [^18^F]NaF uptake	25 (58) 23 (54)	1 (6) 0 (0)	21 (3–172) 48 (2–1022)	.005 .014
Costosternal joint(s) 1–3 Sclerosis/hyperostosis Increased [^18^F]NaF uptake	33 (77) 34 (79)	1 (6) 8 (50)	57 (7–488) 3 (1–12)	<.001 .055
Costochondral transition(s) 1–3 Sclerosis and/or hyperostosis Increased [^18^F]NaF uptake	13 (31) 13 (30)	0 (0) 2 (13)	20 (0.9–424) 3 (0.6–15)	.058 .179

Abbreviations: CNO, chronic nonbacterial osteitis; OR, odds ratio; [^18^F]NaF, sodium fluoride-18; [18F]NaF-PET/CT, sodium fluoride-18 positron emission tomography/computed tomography.

Data are presented as *n* (%).

### Quantitative assessment of CNO lesions on [^18^F]NaF-PET/CT

**Figure 2:**
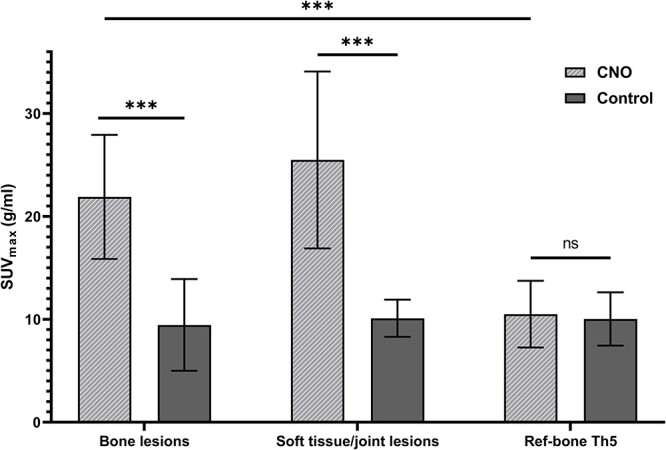
Maximum standardized uptake value (SUV_max_) in g/mL of bone and soft tissue/joint lesions and in reference bone (Ref-bone) Th5 in CNO patients and controls. Values are means ± SD. ^*^^*^^*^p < .01. Abbreviations: CNO, chronic nonbacterial osteitis; ns, nonsignificant.

Quantitative analyses of [^18^F]NaF-PET/CT showed similar uptake for patients and controls in reference bone thoracic vertebra 5 (Th5) (10.5 ± 3.2 g/mL vs 10.0 ± 2.6 g/mL; see Figure 2) and the proximal humerus (3.2 ± 1.2 g/mL and 3.0 ± 1.3 g/mL; data not shown), albeit with larger spread for the latter. In patients, SUV_max_ of bone lesions was markedly higher compared with their reference bone Th5 (mean paired difference, 11.4 g/mL; 95% CI, 9.4–13.5; p < .001), while for controls, SUV_max_ in “bone lesions” and reference bone Th5 was similar. Comparing patients with controls, SUV_max_ of the bone lesions was higher in patients (mean difference, 12.4 g/mL; 95% CI, 9.1–15.8; p < .001), as was SUV_max_ of the soft tissue/joint lesions (mean difference, 15.4 g/mL; 95% CI, 11–19.8; p < .001).

**Figure 3:**
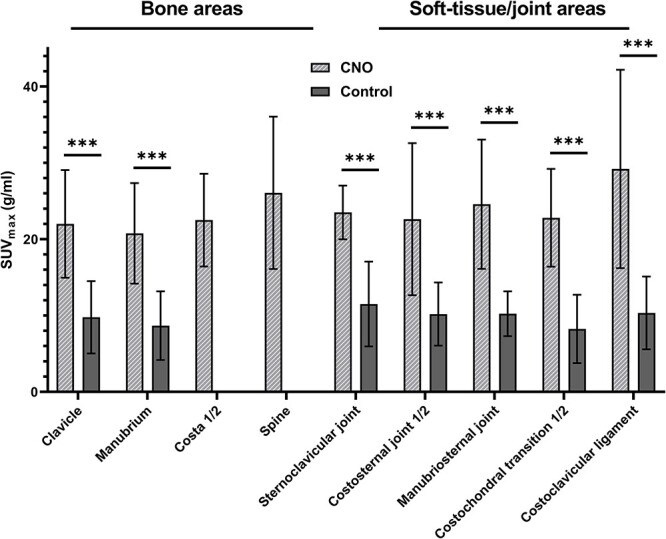
Maximum standardized uptake value (SUV_max_) in g/mL per disease location in patients with CNO and controls. Costa 1/2 and spine were not evaluated for controls. Values are means ± SD. ^*^^*^^*^p < .01. Abbreviation: CNO, chronic nonbacterial osteitis; SUV_max_, standardized maximum uptake value.

SUV_max_ stratified per lesion location is displayed in Figure 3. SUV_max_ was higher in patients at all comparable locations (bone and soft tissue/joint). This also applied to locations for which a qualitative description of [^18^F]NaF uptake (Table 2) could not differentiate between patients and controls (ie, the clavicle, sternoclavicular joint, manubriosternal joint, costosternal joints, and costochondral transitions). Considering bones in patients with CNO, the clavicle, manubrium, and costa 1/2 yielded similar SUV_max_ (mean, 22 ± 7.1, 20.8 ± 6.6, and 22.5 ± 6.6 g/mL, respectively). Spinal locations of CNO showed somewhat higher mean SUV_max_ of 26.1 ± 10.0 g/mL (p = .07 as compared to clavicle/manubrium/costae). With regard to joint and soft tissue involvement in CNO, the costoclavicular ligament proved most active, with a mean of 29.2 ± 13.0 g/mL, also exceeding the mean SUV_max_ of all bones.

In patients with CNO, the number of active bone and soft tissue/joint lesions (defined as demonstrating increased [^18^F]NaF uptake) and their SUV_max_ values were correlated to clinical and biochemical parameters of disease activity (see Table 4). There was a positive, yet subtle to weak correlation between the number of active bone lesions and ESR, serum CTx, serum ALP, and VAS for pain (Spearman’s correlation coefficients of 0.285, 0.306, 0.307, and 0.318, respectively; p < .05 for all). Mean SUV_max_ of soft tissue/joint lesions positively correlated with ESR, but this was not the case for mean SUV_max_ of bone lesions (Spearman’s correlation coefficient, 0.546; p < .002 for the former).

**Table 4:** Correlations between number of active lesions, their SUV_max_, and biochemical and clinical disease activity parameters in patients with CNO.

	**ESR**	**CRP**	**P1NP**	**CTx**	**ALP**	**VAS (pain)**
No. of active bone lesions (range 1–13)	0.285^*^	0.200	−0.002	0.306^*^	0.307^*^	0.318^*^
No. of active soft tissue/joint lesions (range 0–11)	0.160	−0.061	−0.184	0.045	0.001	0.167
Mean SUV_max_ bone lesions	−0.083	−0.196	0.163	0.231	0.105	−0.127
Mean SUV_max_ soft tissue/joint lesions	0.546^*^^*^	0.214	0.088	−0.145	0.255	−0.127

Abbreviations: ALP, alkaline phosphatase; CNO, chronic nonbacterial osteitis; CRP, C-reactive protein; CTx, C-terminal telopeptide of type 1 collagen; ESR, erythrocyte sedimentation rate; P1NP, procollagen 1N-terminal propeptide; SUV_max_, maximum standardized uptake value; VAS, visual analog scale.

^*^p < .05, ^*^^*^p < .01.

The association between mean SUV_max_ in bone and soft tissue/joint lesions and diagnosis of CNO adjusted for symptom duration was evaluated by multivariate logistic regression analysis (Table 5). Both SUV_max_ of bone and soft tissue/joint lesions were associated with CNO (adjusted OR, 1.7; 95% CI, 1.2–2.4 [p = .002]; and OR; 2.1; 95% CI, 1.2–3.7 [p = .011]). SUV_max_ cutoffs optimized for specificity were 17.3 g/mL for bone (sensitivity 81% [95% CI, 65%–91%] and specificity 94% [95% CI, 68%–100%]) and 15.3 g/mL for soft tissue/joint (92% [95% CI, 78%–98%] and 100% [95% CI, 76%–100%], respectively).

**Table 5:** Odds ratios adjusted for symptom duration, cutoff optimized for specificity, and sensitivity and specificity for SUV_max,_ (mean and highest, bone and soft tissue/joint).

	**OR (95% CI) per unit increase** ^**a**^	**Optimal cutoff, g/mL**	**Sensitivity (95% CI)**	**Specificity (95% CI)**
SUV_max_ bone (mean)	1.7 (1.2–2.4)	17.3	81% (65%–91%)	94% (68%–100%)
SUV_max_ soft tissue/joint (mean)	2.1 (1.2–3.7)	15.3	92% (78%–98%)	100% (76%–100%)

Abbreviations: OR, odds ratio; SUV_max_, maximum standardized uptake value.

a Adjusted for symptom duration.

## Discussion

Chronic nonbacterial osteitis is an impactful rheumatic disorder characterized by chronic bone inflammation and long-term hyperostotic transformation. Imaging takes on a pivotal role in diagnosis and disease activity assessment, because clinical and biochemical markers may be nonspecific.^3^ This study evaluated the capacities of [^18^F]NaF-PET/CT in the structural and functional assessment of adult CNO. We first demonstrated that [^18^F]NaF-PET/CT produces high-quality images that well display the structural hallmarks of CNO, as identified in previous studies. We found that features associated with adult CNO included sclerosis and hyperostosis of the manubrium and costae 1–2 and calcification/ankylosis of the costoclavicular ligament. This is in full accordance with prior studies that have described these radiologic abnormalities in adult CNO using [^99m^Tc]Tc-HDP SPECT/CT and MRI.^15–21^

In addition to providing a thorough structural assessment, [^18^F]NaF-PET/CT offers technical and logistical benefits compared with alternative imaging methods. Like [^99m^Tc]Tc-HDP SPECT/CT, [^18^F]NaF-PET/CT conveniently combines CT and nuclear imaging in 1 procedure, but [^18^F]NaF-PET/CT has a shorter incubation and scanning time, higher spatial resolution, and significantly lower radiation exposure due to its pharmacokinetics (total exposure estimated at 209 MBq compared to 672 MBq for [^99m^Tc]Tc-HDP SPECT/CT).^10^^,^^11^^,^^22^ Compared with MRI, the CT component of [^18^F]NaF-PET/CT offers a superior structural evaluation, especially for the anterior chest wall, which is the primary localization of adult CNO, involved in 89% of patients.^3^ In this region, CT yields higher spatial resolution, being less compromised by motion and breathing artefacts. Computed tomography also enables 3D reconstructions, which are necessary to sensitively detect incipient areas of sclerosis, hyperostosis, periosteal new bone formation, ankylosis, and calcifications, all of which inform about disease duration, progression, and severity.^6^^,^^19^^,^^20^^,^^23–25^) Indeed, a recent study performed both MRI and CT of the anterior chest wall in adult patients with CNO, where CT detected a greater number of structural lesions.^26^ Altogether, we argue that [^18^F]NaF-PET/CT is preferred over [^99m^Tc]Tc-HDP SPECT/CT and MRI to provide structural characterization of adult CNO, especially in the anterior chest wall, being a technically easy, fast, and widely available tool that produces high-quality images of the relevant regions.

**Figure 4:**
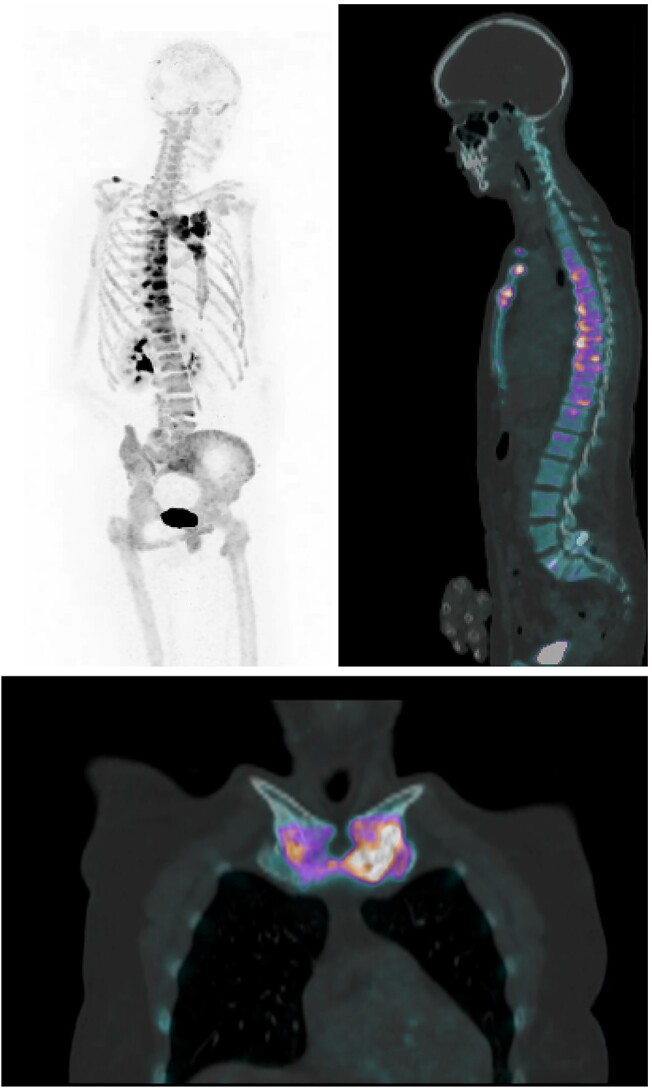
Maximum intensity project (MIP; left upper panel), sagittal (right upper panel), and coronal (lower pane;) views of CNO on [^18^F]NaF-PET/CT in a patient with involvement of the anterior chest wall and thoracic vertebrae. Abbreviations: CNO, chronic nonbacterial osteitis; [18F]NaF-PET/CT, sodium fluoride-18 positron emission tomography/computed tomography.

**Figure 5:**
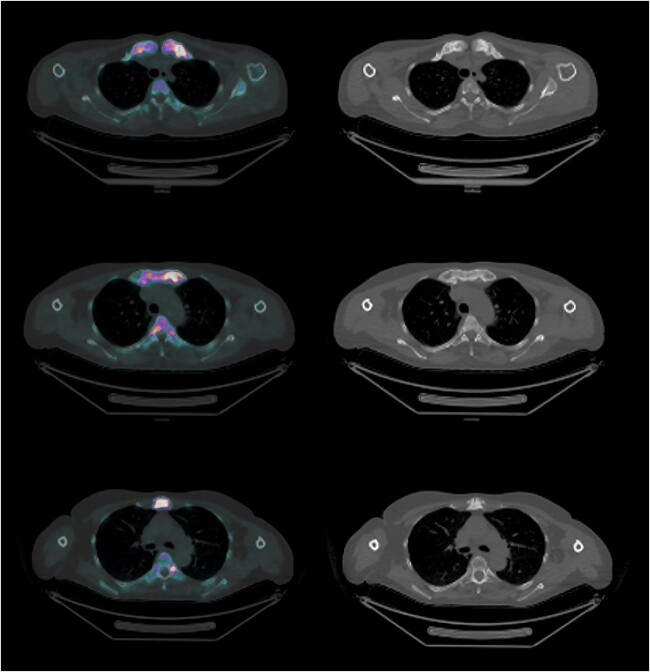
Transversal views of CNO on [^18^F]NaF-PET/CT visualizing clavicular (upper), costosternal (middle), and manubrial (lower) sclerosis, hyperostosis, and increased [^18^F]NaF-uptake. Abbreviations: CNO, chronic nonbacterial osteitis; [18F]NaF-PET/CT, sodium fluoride-18 positron emission tomography/computed tomography.

In addition to structural assessment, [^18^F]NaF-PET/CT offers the advantage of simultaneously providing functional assessment in a single scan, readily yielding quantitative parameters of bone turnover (see Figures 4 and 5 for illustrative imaging slides). As Na^18^F precipitates in young osteoid, the degree of uptake reflects the degree of bone turnover, which is commonly increased as a result of inflammation in diseases like CNO. Indeed, we found markedly higher SUV_max_ values in all evaluated CNO bone and soft tissue/joint lesions as compared with in-patient reference bone and with controls, suggesting that high [^18^F]NaF uptake is associated with inflammatory disease pathology. Also, mean SUV_max_ of soft tissue/joint lesions positively correlated with ESR (*r* = 0.546, p < .01). Hence, quantified [^18^F]NaF-uptake in CNO lesions may be a candidate disease activity biomarker. This would be a major advancement in CNO disease monitoring, as current radiologic techniques have shortcomings for this purpose: [^99m^Tc]Tc-HDP SPECT/CT shows an imprinting pattern of increased isotope uptake and BME is poorly visible on MRI in the presence of sclerosis and hyperostosis.^5^^,^^7^

The highest absolute SUV_max_ values were found in soft tissue/joint lesions, specifically in the costoclavicular ligament (mean, 29.2 ± 13.0 in patients). This finding is of interest, given that these ligaments are known to undergo a calcification process over time, potentially resulting in complete ankylosis in long-term CNO.^20^ Our findings therefore suggest that quantified [^18^F]NaF uptake may not only represent bone turnover but also the process of new bone formation. This property of [^18^F]NaF uptake has been discovered in other diseases characterized by new bone formation as well, such as fibrodysplasia ossificans progressiva^27^, axial spondylarthritis,^28–30^ and psoriatic arthritis.^31^

Our data also add to the ongoing discussion on the primary disease process in CNO and its chronology. It is generally recognized that calcifications of ligaments and joint capsules are prominent features of long-term CNO^17^^,^^32^, which corresponds to the idea that CNO starts with osteitis and affects adjacent joints and ligaments only secondarily.^20^^,^^33^ Contrarily, a landmark radiographic study proposed that the ossification of the costoclavicular ligament isan early phase of the disease^34^, suggesting that the primary pathology in CNO is inflammatory enthesitis rather than osteitis. This idea was confirmed in a recent study in which most inflammatory signs were found at the insertions of the ligaments and joint capsules.^32^ While our cross-sectional data cannot reflect the progression of changes during CNO’s disease course, it remains striking that soft tissue/joint lesions demonstrate the highest SUV_max_ values, and that the SUV_max_ in soft tissue/joint lesions positively correlates with ESR. In that sense, our results modestly support the hypothesis of inflammatory enthesitis being a primary, if not key, disease process in CNO.

The clinical impact of our findings is substantial. First, [^18^F]NaF-PET/CT bone turnover parameters form an important advancement in disease activity assessment in adult CNO, which is currently highly difficult as clinical and biochemical measures are nonspecific.^3^ [^18^F]NaF-PET/CT provides radiologic biomarkers allowing for accurate and objective disease monitoring, which can support clinicians and patients in understanding the disease course and making better-informed therapeutic decisions. Second, [^18^F]NaF-PET/CT visualizes the early stages of new bone formation in CNO, an irreversible and detrimental disease process associated with poor outcomes due to mechanical complications. Early detection of this process enables proactive and potentially more aggressive treatment to prevent permanent skeletal damage, which is highly relevant to long-term disease management.^21^^,^^34^

Our study has several limitations. First, diagnosis of CNO was based on the assessment of a multidisciplinary expert team. While there are published criteria for CNO available, these were devised for pediatric patients^35^^,^^36^ or pertain to the broad entity of SAPHO syndrome, which can include also patients without the core disease characteristic of osteitis.^3^^,^^37^^,^^38^ In addition, none of the criteria sets have been externally validated. We therefore use expert-based diagnosis until better criteria are available. Even so, all of our patients also retrospectively fulfilled the Jansson criteria for CNO, allowing for comparison with other cohorts.

Second, the diagnosis of CNO—whether expert-based or criteria-based—relies in large part on radiologic features, specifically hyperostosis. Therefore, it is not surprising that hyperostosis appears associated with CNO, as it likely prompts diagnosis in the first place. However, our data additionally inform about other, less conventional features that we identify to be associated with CNO, including SUV_max_ values. These were shown to be associated with CNO but could not have guided diagnosis as they were generated retrospectively for the purpose of this study and are not necessarily correlated with steering features like hyperostosis.^21^ Third, the sample size of the control group was small, limiting the power of our analyses and inducing substantial imprecision as reflected in the wide CIs for, particularly, the ORs of radiologic features. Due to the limited sample size, these radiologic features could also not meaningfully be adjusted for symptom duration, which would have been of interest as well. Also, our qualitative assessments were made by 1 radiologist and 1 nuclear medicine physician. However, both have many years of experience, also with CNO specifically, and we used a systematic reporting format to ensure fair reproducibility. A strength of this study is the choice of SUV_max_ as an imaging parameter, which is recognized for its reproducibility between observers and stability across scanners and time, giving our results marked clinical outreach.^39^^,^^40^

In sum, [^18^F]NaF-PET/CT is a promising imaging tool for adult CNO, especially for the anterior chest wall. It allows for detailed structural evaluation of bone and soft tissue/joint lesions and functional evaluation by yielding quantitative data representing bone turnover and new bone formation. Further studies should investigate the application and clinical utility of quantified [^18^F]NaF uptake as a novel biomarker for disease activity in CNO.

## Funding

This research received support from ReumaNederland.

## Conflicts of interest

The authors declare no competing interests pertaining to the current study.

## Data availability

The data that support the findings of this study are available from the corresponding author upon reasonable request.

## Supplementary Material

S1_Standardized_reporting_format_revised_without_track_changes_ziad007

## References

[1] Buch K , Thuesen ACB, Brons C, Schwarz P. Chronic non-bacterial osteomyelitis: a review. Calcif Tissue Int. 2019;104(5):544–553. 10.1007/s00223-018-0495-0. 30456556

[2] Rukavina I . SAPHO syndrome: a review. J Child Orthop. 2015;9(1):19–27. 10.1007/s11832-014-0627-7. 25585872 PMC4340847

[3] Leerling A , Dekkers O, Appelman-Dijkstra N, Winter E. Clinical and therapeutic diversity in adult chronic nonbacterial osteomyelitis (CNO) of the sternocostoclavicular region: a meta-analysis. Rheumatology (Oxford). 2023;62(2):512–522. 10.1186/s13023-023-02831-1. PMC9891421 35961032

[4] Carroll MB . Sternocostoclavicular hyperostosis: a review. Ther Adv Musculoskelet Dis. 2011;3(2):101–110. 10.1177/1759720X11398333. 22870470 PMC3382681

[5] Li C , Wang L, Wu N, et al. A retrospective study of bone scintigraphy in the follow-up of patients with synovitis, acne, pustulosis, hyperostosis, and osteitis syndrome: is it useful to repeat bone scintigraphy for disease assessment? Clin Rheumatol. 2019;39(4):1305–1314. 10.1007/s10067-019-04864-z. 31858336

[6] Gu Z , Li C, Xu W, et al. Disease activity in patients with synovitis, acne, pustulosis, hyperostosis, and osteitis (SAPHO) syndrome: the utility of the SPARCC MRI scoring system for assessment of axial spine involvement. Clin Exp Rheumatol. 2021;39(6):1291–1297. 10.55563/clinexprheumatol/hhdeuu. 33427614

[7] Chaturvedi A . Pediatric skeletal diffusion-weighted magnetic resonance imaging, part 2: current and emerging applications. Pediatr Radiol. 2021;51(9):1575–1588. 10.1007/s00247-021-05028-5. 34018037

[8] Velez EM , Desai B, Jadvar H. Treatment response assessment of skeletal metastases in prostate cancer with (18)F-NaF PET/CT. Nucl Med Mol Imaging. 2019;53(4):247–252. 10.1007/s13139-019-00601-1. 31456857 PMC6694323

[9] van der Bruggen W , Hagelstein-Rotman M, de Geus-Oei LF, et al. Quantifying skeletal burden in fibrous dysplasia using sodium fluoride PET/CT. Eur J Nucl Med Mol Imaging. 2020;47(6):1527–1537. 10.1007/s00259-019-04657-1. 31875244

[10] Iagaru A , Young P, Mittra E, Dick DW, Herfkens R, Gambhir SS. Pilot prospective evaluation of 99mTc-MDP scintigraphy, 18F NaF PET/CT, 18F FDG PET/CT and whole-body MRI for detection of skeletal metastases. Clin Nucl Med. 2013;38(7):e290–e296. 10.1097/RLU.0b013e3182815f64. 23455520

[11] Sarikaya I , Elgazzar AH, Sarikaya A, Alfeeli M. Normal bone and soft tissue distribution of fluorine-18-sodium fluoride and artifacts on 18F-NaF PET/CT bone scan: a pictorial review. Nucl Med Commun. 2017;38(10):810–819. 10.1097/MNM.0000000000000720. 28777220

[12] von Elm E , Altman DG, Egger M, et al. Strengthening the Reporting of Observational Studies in Epidemiology (STROBE) statement: guidelines for reporting observational studies. BMJ. 2007;335(7624):806–808. 10.1136/bmj.39335.541782.AD. 17947786 PMC2034723

[13] Mendoza T , Mayne T, Rublee D, Cleeland C. Reliability and validity of a modified brief pain inventory short form in patients with osteoarthritis. *Eur J* Pai*n*. 2006;10(4):353–361. 10.1016/j.ejpain.2005.06.002. 16051509

[14] Haldane JB . The estimation and significance of the logarithm of a ratio of frequencies. Ann Hum Genet. 1956;20(4):309–311. 10.1111/j.1469-1809.1955.tb01285.x. 13314400

[15] Cao Y , Li C, Yang Q, et al. Three patterns of osteoarticular involvement in SAPHO syndrome: a cluster analysis based on whole body bone scintigraphy of 157 patients. Rheumatology. 2019;58(6):1047–1055. 10.1093/rheumatology/key415. 30624750

[16] Gao S , Deng X, Zhang L, Song L. The comparison analysis of clinical and radiological features in SAPHO syndrome. Clin Rheumatol. 2021;40(1):349–357. 10.1007/s10067-020-05187-0. 32504191

[17] Himuro H , Kurata S, Nagata S, et al. Imaging features in patients with SAPHO/CRMO: a pictorial review. Jpn J Radiol. 2020;38(7):622–629. 10.1007/s11604-020-00953-1. 32356235

[18] Ramautar A , Navas CA, Winter E, et al. Defining the radiological diagnostic criteria for adult chronic non-bacterial osteomyelitis of the sternocostoclavicular region (CNO/SCCH). J Bone Miner Res Plus. (in press).

[19] Earwaker JWS , Cotten A. SAPHO: syndrome or concept? Imaging findings. Skelet Radiol. 2003;32(6):311–327. 10.1007/s00256-003-0629-x. 12719925

[20] Jurik AG , Klicman RF, Simoni P, Robinson P, Teh J. SAPHO and CRMO: the value of imaging. Semin Musculoskelet Radiol. 2018;22(02):207–224. 10.1055/s-0038-1639469. 29672809

[21] Depasquale R , Kumar N, Lalam RK, et al. SAPHO: what radiologists should know. Clin Radiol. 2012;67(3):195–206. 10.1016/j.crad.2011.08.014. 21939963

[22] Arvola S , Jambor I, Kuisma A, et al. Comparison of standardized uptake values between (99m)Tc-HDP SPECT/CT and (18)F-NaF PET/CT in bone metastases of breast and prostate cancer. Eur J Nucl Med Mol Imaging. 2019;9(1):6. 10.1186/s13550-019-0475-z. PMC6346696 30680469

[23] Zhao Y , Chauvin NA, Jaramillo D, Burnham JM. Aggressive therapy reduces disease activity without skeletal damage progression in chronic nonbacterial osteomyelitis. J Rheumatol. 2015;42(7):1245–1251. 10.3899/jrheum.141138. 25979712

[24] Raptis CA , Ludwig DR, Hammer MM, et al. Building blocks for thoracic MRI: challenges, sequences, and protocol design. J Magn Reson Imaging. 2019;50(3):682–701. 10.1002/jmri.26677. 30779459

[25] Alfudhili K , Masci PG, Delacoste J, et al. Current artefacts in cardiac and chest magnetic resonance imaging: tips and tricks. Br J Radiol. 2016;89(1062):20150987. 10.1259/bjr.20150987. 26986460 PMC5258161

[26] Andreasen CM , Jurik AG, Deleuran BW, et al. Pamidronate in chronic non-bacterial osteomyelitis: a randomized, double-blinded, placebo-controlled pilot trial. Scand J Rheumatol. 2020;49(4):312–322. 10.1080/03009742.2020.1724324. 32484386

[27] Botman E , Raijmakers P, Yaqub M, et al. Evolution of heterotopic bone in fibrodysplasia ossificans progressiva: an [(18)F]NaF PET/CT study. Bone. 2019;124:1–6. 10.1016/j.bone.2019.03.009. 30858149

[28] Tan S , Wang R, Ward MM. Syndesmophyte growth in ankylosing spondylitis. Curr Opin Rheumatol. 2015;27(4):326–332. 10.1097/BOR.0000000000000179. 26002023 PMC4478446

[29] van Tubergen A , Ramiro S, van der Heijde D, Dougados M, Mielants H, Landewe R. Development of new syndesmophytes and bridges in ankylosing spondylitis and their predictors: a longitudinal study. Ann Rheum Dis. 2012;71(4):518–523. 10.1136/annrheumdis-2011-200411. 21989544

[30] Bruijnen STG , Verweij NJF, van Duivenvoorde LM, et al. Bone formation in ankylosing spondylitis during anti-tumour necrosis factor therapy imaged by 18F-fluoride positron emission tomography. Rheumatology (Oxford). 2018;57(4):631–638. 10.1093/rheumatology/kex448. 29329443 PMC5888961

[31] de Jongh J , Hemke R, Zwezerijnen GJC, et al. (18)F-sodium fluoride PET-CT visualizes both axial and peripheral new bone formation in psoriatic arthritis patients. Eur J Nucl Med Mol Imaging. 2023;50(3):756–764. 10.1007/s00259-022-06035-w. 36370181 PMC9852163

[32] Yu M , Cao Y, Li J, et al. Anterior chest wall in SAPHO syndrome: magnetic resonance imaging findings. Arthritis Res Ther. 2020;22(1):216. 10.1186/s13075-020-02309-6. 32928273 PMC7491189

[33] Xu W , Li C, Zhao X, et al. Whole-spine computed tomography findings in SAPHO syndrome. J Rheumatol. 2017;44(5):648–654. 10.3899/jrheum.161075. 28250144

[34] Sonozaki H , Azuma A, Okai K, et al. Clinical features of 22 cases with ``inter-sterno-costo-clavicular ossification''. A new rheumatic syndrome. Arch Orthop Trauma Surg. 1979;95(1–2):13–22. 10.1007/BF00379164. 118719

[35] Jansson AF , Muller TH, Gliera L, et al. Clinical score for nonbacterial osteitis in children and adults. Arthritis Rheum. 2009;60(4):1152–1159. 10.1002/art.24402. 19333943

[36] Roderick MR , Shah R, Rogers V, Finn A, Ramanan AV. Chronic recurrent multifocal osteomyelitis (CRMO)—advancing the diagnosis. Pediatr Rheumatol Online J. 2016;14(1):47. 10.1186/s12969-016-0109-1. 27576444 PMC5006369

[37] Kahn MF , Khan MA. The SAPHO syndrome. Baillieres Clin Rheumatol. 1994;8(2):333–362. 10.1016/S0950-3579(94)80022-7. 8076391

[38] Kahn MF , Chamot AM. SAPHO syndrome. *Rheum Dis Clin N* A*m*. 1992;18(1):225–246. 10.1016/S0889-857X(21)00720-1. 1532859

[39] Lindholm H , Staaf J, Jacobsson H, Brolin F, Hatherly R, Sanchez-Crespo A. Repeatability of the maximum standard uptake value (SUVmax) in FDG PET. Mol Imaging Radionucl Ther. 2014;23(1):16–20. 10.4274/Mirt.76376. 24653930 PMC3957966

[40] Lin C , Bradshaw T, Perk T, et al. Repeatability of quantitative F-NaF PET: a Multicenter study. J Nucl Med. 2016;57(12):1872–1879. 10.2967/jnumed.116.177295. 27445292 PMC6952054

