# Supplementary material for: 18F-Sodium fluoride PET-CT visualizes disease activity in chronic nonbacterial osteitis in adults
Source: JBMR Plus. 2024 Jan 4;8(2):ziad007. doi: 10.1093/jbmrpl/ziad007 (PMC10945721; doi:10.1093/jbmrpl/ziad007)
Supplement: S1_Standardized_reporting_format_revised_without_track_changes_ziad007 [file s1_standardized_reporting_format_revised_without_track_changes_ziad007.docx]

## Supplementary data (S1)

**Definitions of abnormalities as scored in standardized reporting format:**

1. **Sclerosis:** increased subchondral bone density AND/OR increased intramedullary bone mineral density (thus away from the subchondral bone plate) compared to a reference bone.

1. **Hyperostosis:** increased diameter of the bone compared to normal diameter on the healthy side or general normal size in the case of bilateral changes AND/OR abnormal thickening of the cortex AND/OR soft tissue ossifications (of the SC and/or costosternal joints) with/without associated ankylosis.

1. **Erosion:** focal cortical defect at the joint facet (usually without sclerotic margin) AND/OR irregular contours of the articular surface of the bone (“fluffy aspect”).

**Standardized report:**

First impression: pharmacon biodistribution, evaluability of the scan
Sufficiently assessable: yes/no

Clavicle:

- Sclerosis:
  - Left/right/bilateral
  - Severity: mild, moderate with visible trabecularisation, moderate without visible trabecularisation, severe
  - Area: small, intermediate, large
- Hyperostosis
  - Left/right/bilateral
  - Severity: mild, severe
- Increased Na^18^F uptake:
  - Left/right/bilateral
  - Severity: moderate, severe

Sternoclavicular joint:

- Soft tissue swelling
  - Left/right/bilateral
  - Severity: mild, severe
- Calcifications of soft tissue: yes/no
- Erosion: yes/no
- Increased Na^18^F uptake:
  - Left/right/bilateral
  - Severity: moderate, severe

Costoclavicular ligament:

- Calcification or ankylosis
  - Left/right/bilateral
  - Severity: mild, severe
- Increased Na^18^F uptake:
  - Left/right/bilateral
  - Severity: moderate, severe

Manubriosternal joint

- Sclerosis: yes/no
- Erosions: yes/no
- Hyperostosis: yes/no
- Ankylosis: yes/no
  - If yes, appearance of congenital ankylosis or resulting from inflammation?
- Increased Na^18^F uptake:
  - Left/right/bilateral
  - Severity: moderate, severe

Costosternal area:

- Sclerosis and hyperostosis
  - Left/right/bilateral
  - Level
- Increased Na^18^F uptake:
  - Left/right/bilateral
  - Severity: moderate, severe

Costochondral transitions:

- Sclerosis and hyperostosis
  - Left/right/bilateral
  - Level
- Increased Na^18^F uptake:
  - Left/right/bilateral
  - Severity: moderate, severe

Mandible:

- Sclerosis: yes/no, left/right/bilateral
- Hyperostosis: yes/no, left/right/bilateral
- Increased Na^18^F uptake:
  - Left/right/bilateral
  - Severity: moderate, severe

Spine:

- Sclerosis
  - Level
- Hyperostosis: yes/no
- Ankylosis: yes/no
- Increased Na^18^F uptake:
  - Severity: moderate, severe
  - Level
